# Nontoxigenic *tox*-bearing *Corynebacterium ulcerans* Infection among Game Animals, Germany

**DOI:** 10.3201/eid2003.130423

**Published:** 2014-03

**Authors:** Tobias Eisenberg, Peter Kutzer, Martin Peters, Andreas Sing, Matthias Contzen, Jörg Rau

**Affiliations:** Landesbetrieb Hessisches Landeslabor, Gießen, Germany (T. Eisenberg); Landeslabor Berlin-Brandenburg, Frankfurt (Oder), Germany (P. Kutzer);; Chemisches und Veterinäruntersuchungsamt Westfalen, Standort Arnsberg, Germany (M. Peters);; Bayerisches Landesamt für Gesundheit und Lebensmittelsicherheit, Oberschleißheim, Germany (A. Sing);; Chemisches und Veterinäruntersuchungsamt Stuttgart, Fellbach, Germany (M. Contzen, J. Rau)

**Keywords:** *Corynebacterium ulcerans*, *Sus scrofa*, *Capreolus capreolus*, wildlife, zoonoses, diphtheria toxin, diphtheria, pseudotuberculosis, toxigenic, nontoxigenic, Germany, bacteria

## Abstract

*Corynebacterium ulcerans* may cause diphtheria in humans and caseous lymphadenitis in animals. We isolated nontoxigenic *tox*-bearing *C. ulcerans* from 13 game animals in Germany. Our results indicate a role for game animals as reservoirs for zoonotic *C. ulcerans*.

The *Corynebacterium* species *C. diphtheriae*, *C. ulcerans*, and *C. pseudotuberculosis* form the *C. diphtheriae* group, as shown by 16S rRNA gene sequence analysis (*1*). Strains of this group carrying lysogenic β-corynephages might produce the *tox*-encoded diphtheria toxin (DT) (*2*). Moreover, *C. ulcerans* and *C. pseudotuberculosis* may produce phospholipase D, the major virulence factor involved in caseous lymphadenitis, which is a disease that mainly affects sheep, goats, and horses (*3*).

From a public health perspective, diphtheria is the most critical human disease attributed to coryneform bacteria (*3*). In recent years, cases of diphtheria caused by *C. ulcerans* have outnumbered those caused by *C. diphtheriae* (*4*). *C. diphtheriae* carriage is nearly exclusively restricted to humans; *C. ulcerans* is a zoonotic pathogen and has been found in various animal species that have contact with humans (*5*). *C. ulcerans* is most closely related to *C. pseudotuberculosis*, and distinction between these species is often difficult when using standard bacteriological methods (*5*). The aim of this study was to comprehensively characterize 13 *C. ulcerans* strains isolated from game animals in Germany.

## The Study

Strains of *C. ulcerans* were isolated during routine bacteriological investigations in conjunction with necropsies of wild animals that were found dead or that had suspicious lesions during 1997–2013. Isolates of coryneform bacteria were subjected to conventional biochemical tests (*3*), and were evaluated after prolonged incubation at 37°C for as long as 14 days. For further characterization, commercial tests API Coryne and VITEK2-compact with cards for coryneform bacteria and corynebacteria and anaerobes (bioMérieux, Nürtingen, Germany) were used according to the manufacturer’s instructions. 

We conducted the reverse CAMP test by using *Staphylococcus aureus* American Type Culture Collection (ATCC [Manassas, VA, USA]) 25923 and the CAMP test by using *Rhodococcus equi* ATCC 33701 according to standard procedures on Columbia sheep blood agar (Oxoid, Wesel, Germany) (*3*). We determined DT production using a modified Elek test (*6*); we used *C. diphtheriae* NCTC 10648 and *C. diphtheriae* NCTC 10356 as positive and negative controls, respectively; and performed a cytotoxicity assay using Vero cells (*7*). The *rpoB* and *tox* genes were partially amplified by using primer pairs C2700F/C3130R and DT1/DT2, respectively, as described (*5*).

**Figure 1:**
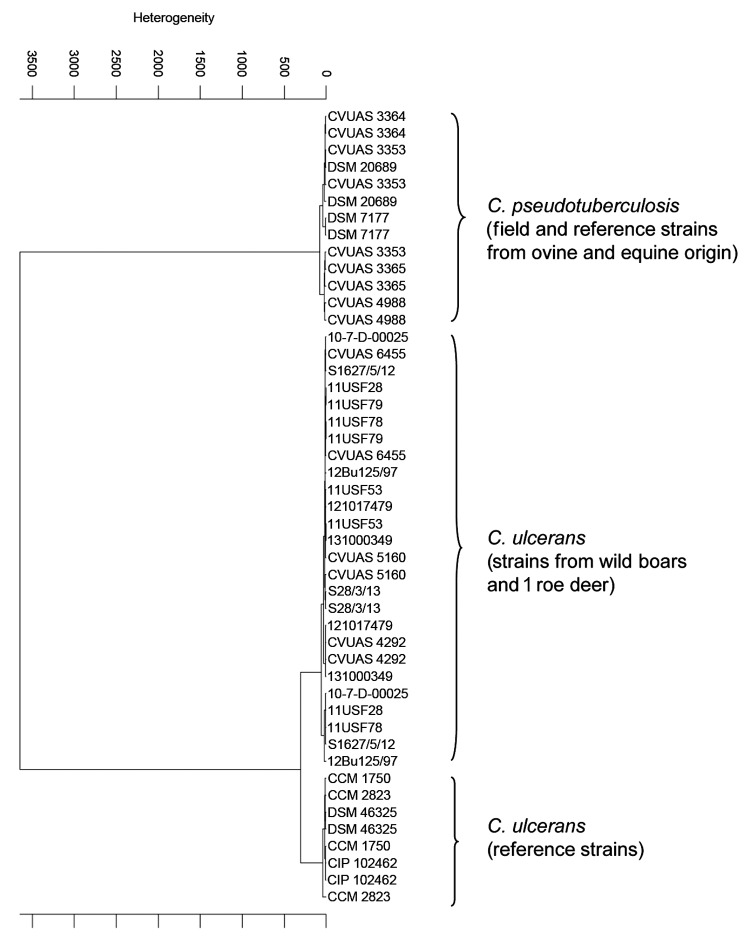
Cluster analysis of respective spectra obtained by Fourier-transform infrared-spectroscopy by using OPUS Software version 4.2 (BrukerOptics, Ettlingen, Germany). In each case, 2 infrared spectra of isolates from game animals and a selection of several *Corynebacterium ulcerans* and *C. pseudotuberculosis* strains were used for calculation by using the Ward algorithm. The dendrogram depicts the arrangement of isolates in groups according to their spectral differences.

PCR products were purified for sequence analysis by using the Double Pure Combi Kit (Bio&SELL, Nürnberg, Germany). Both strands of the *rpoB* and *tox* PCR products were sequenced by Microsynth (Balgach, Switzerland) by using the amplification primers. Sequence analysis was performed by using the BLAST (http://blast.ncbi.nlm.nih.gov/Blast.cgi) sequence analysis tool. Additionally, coryneform isolates in which *C. ulcerans* was suspected were analyzed by using Matrix-assisted laser desorption-ionization time-of-flight mass spectrometry (MALDI-TOF MS) and by using Biotyper version 3.3.1.0 (BrukerBiotyper; BrukerDaltonics, Bremen, Germany). The database used (DB 4613) comprised spectra from 71 *Corynebacterium* species including *C. diphtheriae*, *C. ulcerans*, and *C. pseudotuberculosis*. For Fourier-transform infrared (FT-IR) spectroscopy, bacterial isolates were harvested and prepared as described (*5*). IR spectra were recorded by using an FT-IR spectrometer (Tensor 27 with High Throughput Screening eXTension HTS-XT module) and OPUS software version 4.2 (BrukerOptics, Ettlingen, Germany). IR spectra of isolates from game animals and selected *C. ulcerans* and *C. pseudotuberculosis* strains were compared by cluster analysis by using the second derivation of vector normalized spectra (*8*). The dendrogram obtained depicts the arrangement of isolates in groups according to their spectral differences (Figure 1).

**Figure 2:**
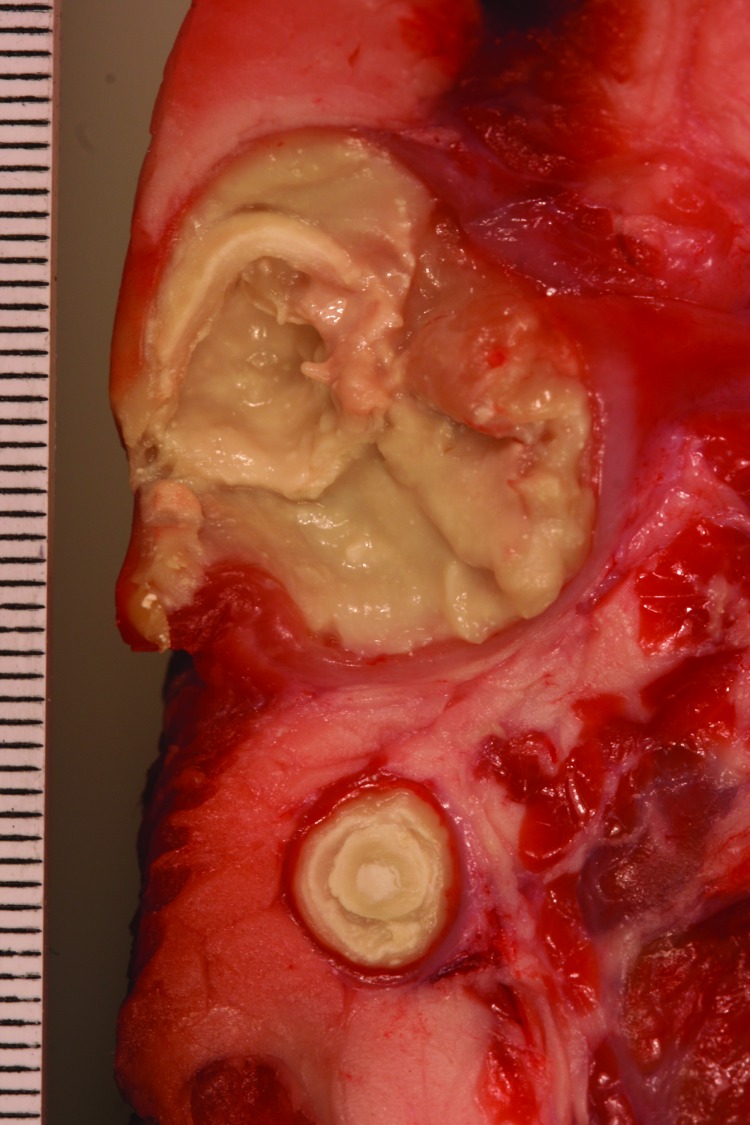
Pseudotuberculosis-like caseous abscesses caused by *Corynebacterium ulcerans* in wild boar S28/3/13. Scale is shown in millimeters.

In total, 13 strains of *C. ulcerans* were isolated from 12 wild boars and 1 roe deer in 4 states of Germany (Table 1). The bacteria grew from >1 delimited pseudotuberculosis-like caseous abscess, arranged in concentric layers and ranging from 0.1 to 10 cm in diameter (Figure 2). All strains had positive reactions by using a traditional CAMP test inoculated with *R. equi* and a reverse CAMP test inoculated with *S. aureus*, indicating phospholipase D activity (*3*).

**Table 1:** Origin of nontoxigenic *tox*-bearing *Corynebacterium ulcerans* field strains among game animals and gross pathology results from necropsies, Germany

Case no.	Isolate ID	Year of isolation	State/district of origin	Host species	Circumstances of death; gross pathology results	Source
1	Bu125/97	1997	North Rhine-Westphalia/ Siegen-Wittgenstein	Wild boar	Meat-inspection; lamellar lymph node abscess	This study
2	CVUAS 4292	2009	Baden Wuerttemberg/ Enz	Wild boar	Found dead; multiple lamellar lymph node abscesses; multiple hypertrophic lymphangitis	(*5*)
3	CVUAS 5160	2009	Baden Wuerttemberg/ Main-Tauber	Wild boar	Shot; superficial cervical lymph nodes greatly enlarged; abscess of *Ln. mandibularis*	(*5*)
4	CVUAS 6455	2010	Baden Wuerttemberg/ Aalen	Roe deer	Moribund; grapefruit-sized abscess of or near left *Ln. cervicalis superficialis*	(*8*)
5	10–7-D-00025	2010	Hesse/ Lahn-Dill	Wild boar	Shot; female; lamellar thoracic plum-sized lymph node abscess	This study
6	11USF28	2011	Brandenburg/ Havelland	Wild boar	Found dead; male, ≈2 y old; subcutaneous abscess	This study
7	11USF53	2011	Brandenburg/ Havelland	Wild boar	Shot; female, ≈3 y old; lung abscess	This study
8	11USF78	2011	Brandenburg/ Havelland	Wild boar	Shot; female, ≈1 y old; subcutaneous abscess	This study
9	11USF79	2011	Brandenburg/ Havelland	Wild boar	Shot; male, ≈1 y old; subcutaneous abscess	This study
10	121017479	2012	Hesse/ Marburg	Wild boar	Shot; some milium- to pea-sized solid grayish abscesses with dystrophic central calcification in diaphragmatic peritoneum	This study
11	S1627/5/12	2012	North Rhine-Westphalia/ Siegen-Wittgenstein	Wild boar	Shot; 1 y old; multiple lamellar abscesses in cervical and pulmonal lymph nodes	This study
12	S28/3/13	2013	Hesse/ Bad Hersfeld	Wild boar	Shot; 2 y old; isolate from teat abscess; multiple lamellar abscesses in cervical lymph nodes	This study
13	131000349	2013	Hesse/ Odenwald	Wild boar	Found dead; female, ≈1 y old; some cherry-sized subcutaneous lymph node abscesses	This study

Conventional biochemical tests showed congruent results for catalase activity, urea hydrolysis, and glucose acidification (positive) and for esculin hydrolysis and nitrate reduction (negative). Additional reactions and API and VITEK test results are shown in Table 2. All isolates were nontoxigenic *tox*-bearing (NTTB) strains as shown by positive *tox*-PCR, and negative Elek test and Vero cell cytotoxicity results. Partial *rpoB* and partial *tox* sequences for all 13 isolates were identical to those submitted to GenBank for *C. ulcerans* strain CVUAS 4292 (accession nos. GU818735 and GU818742, respectively [*5*]).

**Table 2:** Variable biochemical characteristics of API Coryne and VITEK2-compact profiles(bioMérieux, Nürtingen, Germany) of 13 *Corynebacterium ulcerans* field strains from game animals, Germany*

Isolate ID	Ability of isolate to metabolize carbohydrate					API Coryne profile (interpretation/% ID)	VITEK2 CBC profile (interpretation/%)	VITEK2 ANC profile (interpretation)†
	Maltose	Mannitol	Sucrose	Trehalose	Xylose			
Bu125/97	−	−	−	+	−	0 011 324 (Cps/99.5)	01030140402010 (Cps/96)	2123020000405 (Cul, Cje)
CVUAS 4292	+	−	+	+	+	0 041 725 (invalid)	01030140406010 (Cps, Cdi/ -)	2123020000405 (Cul, Cje)
CVUAS 5160	−	+	−	+	+	0 001 304 (Cps/97.5)	01030140402010 (Cps/ 96)	2123020000405 (Cul, Cje/)
CVUAS 6455	−	+	+	+	−	5 153 325 (invalid)	01030140402010 (Cps/ 96)	2123020000405 (Cul, Cje)
10–7-D-00025	−	−	−	+	−	0 011 324 (Cps/99.5)	01030140402010 (Cps/ 96)	2123020000405 (Cul, Cje)
11USF28	−	+	−	+	+	0 011 324 (Cps/99.5)	01030140402010 (Cps/ 96)	2123020000405 (Cul, Cje)
11USF53	−	−	−	+	−	0 011 324 (Cps/99.5)	01030140402010 (Cps/ 96)	2123020000405 (Cul, Cje)
11USF78	−	−	−	+	−	0 011 324 (Cps/99.5)	01030140402010 (Cps 96)	2123020000405 (Cdi, Cje)
11USF79	+	−	−	+	−	0 011 324 (Cps/99.5)	01030140402010 (Cps/ 96)	2123020000405 (Cul, Cje)
121017479	−	−	−	−	−	0 011 324 (Cps/99.5)	01430140402010 (Cps/ 92)	2123020000405 (Cul, Cje)
S1627/5/12	−	−	−	+	−	0 001 324 (Cps/99.9)	01030140402010 (Cps/ 96)	2123020000405 (Cul, Cje)
S28/3/13	−	−	−	+ (weak)	+	0 111 324 (Cps/92.7, Cul/7.2)	01020140402010 (Cps, Cma/ -)	2123020000405 (Cul, Cje)
131000349	−	−	−	+ (weak)	+	0 011 324 (Cps/99.5)	01030140402010 (Cps/ 96)	2123020000405 (Cul, Cje)

*% ID, probability of identification according to the evaluation by the manufacturer; CBC, coryneform bacteria; ANC, *Corynebacterium* and anaerobes; Cps, *C. pseudotuberculosis*; Cul, *C. ulcerans*; Cdi, *C. diphtheriae*; Cje, *C. jeikeium*; Cma, *C. macginleyi*. †Probability of identification was not provided by the manufacturer for cases in which test results identified 2 organisms.

By using MALDI-TOF MS, all isolates were identified to the species level as *C. ulcerans* because they had score levels of 2.0–2.2. The comparison of the IR spectra of the 13 strains from game animals with a collection of reference strains showed a clear separation in 2 main branches for the 2 species *C. pseudotuberculosis* and *C. ulcerans* (Figure 1). Inside the *C. ulcerans* branch, all isolates from game animals clustered compactly together and were closely adjacent to a group of spectra formed by reference strains from humans.

## Conclusions

With respect to its zoonotic potential, *C. ulcerans* is one of the most notable members of the genus and was referred to as an emerging pathogen in 2011 (*9*). Numerous reports state there is zoonotic potential for contact with companion or farm animals, but proven transmission of *tox*-positive *C. ulcerans* strains is documented for only 4 cases, involving 2 dogs, 1 cat, and 1 pig (*8*).

Limited information is available concerning *C. ulcerans* infection in wild animals. To our knowledge, 3 reports regarding *tox*-positive *C. ulcerans* infection in wildlife have been published: 1 involved 2 European otters from 2 widely separated regions within the United Kingdom (*10*), and the other 2 reports described NTTB strains in 2 wild boars in 1 report and 1 roe deer in the other report; these 3 cases were in the same area of Germany (*5*,*8*). An additional report on *C. ulcerans* with unknown toxigenicity in wildlife pertains to an outbreak among 350 squirrels from Canada, 63 of which had clinical disease (*11*).

Here, we provide comprehensive data on 13 NTTB *C. ulcerans* strains from game animals in Germany. The finding of infected game in the center of Middle Europe suggests an even wider occurrence and distribution in other European countries. Misdiagnoses of *C. ulcerans* isolates as *C. pseudotuberculosis* in the past because of similar pathology and similar phenotype cannot be excluded. Our finding of *C. ulcerans* in a wild boar specimen from 1997 could indicate that this pathogen has not only recently infected wildlife.

As also shown in this study, biochemical differentiation between *C. ulcerans* and *C. pseudotuberculosis* might be problematic, and basic conventional tests may not properly discriminate between the 2 species (*3*). By using the standardized systems API Coryne and VITEK2-compact for coryneform bacteria, erroneous identification was made of most isolates (10 and 11 cases, respectively) from game animals as *C. pseudotuberculosis*. For correct understanding of epidemiology and host range and for unequivocal determination of the involved pathogen to species level, additional methods such as FT-IR and MALDI-TOF MS or DNA sequencing should be used. Because partial *rpoB* sequencing is more discriminatory than 16S rDNA sequencing, a cutoff value of ≤95% similarity proved suitable for species identification within *Corynebacterium* (*12*) and also clearly enabled species identification in this study. Furthermore, partial *tox*- and *rpoB*-gene sequencing demonstrated a very close relationship between the 13 strains because no variations in these sequences were found (*8*).

Concerning the zoonotic potential for *C. ulcerans* strains from wildlife, there is no information available. With respect to wild boars infected with *C. ulcerans*, however, it is noteworthy that 3 diphtheria cases occurred in humans who had occupational contact with pigs (*13*,*14*).

Lack of DT expression in *tox*-positive strains has been described (*7*). Nevertheless, it can be expected that DT-producing *C. ulcerans* strains might occur in game animals, providing a reservoir for this microorganism. Because the *C. diphtheriae* and *C. ulcerans* DT sequences are quite similar, it might be reasonable to offer diphtheria toxoid vaccination to persons who have direct contact with game animals to prevent diphtheria-like illness caused by *tox*-positive *C. ulcerans* (*4*).
